# Mediators of Inflammation in Bone Physiology and Diseases

**DOI:** 10.1155/2022/9767408

**Published:** 2022-04-13

**Authors:** Drenka Trivanović, Michaela Tencerova, Marietta Herrmann, Alanna C. Green, Petar Milovanović

**Affiliations:** ^1^IZKF Group Tissue Regeneration in Musculoskeletal Diseases, University Hospital Wuerzburg & Bernhard-Heine-Center for Locomotion Research, University of Wuerzburg, Wuerzburg, Germany; ^2^Institute of Physiology of the Czech Academy of Sciences, Prague, Czech Republic; ^3^Department of Oncology & Metabolism, University of Sheffield, Sheffield, UK; ^4^Institute of Anatomy, Faculty of Medicine, University of Belgrade, Belgrade, Serbia

Complex crosstalk between immune cells and cells involved in skeletal renewal is essential for bone health. Bone tissue formation and resorption are governed by the inflammation initiated by endogenous or exogenous stimuli, as well as local and systemic metabolism and the aging process. This special issue is focused on the topic of inflammation in bone physiology contributing to the novel findings in the osteoimmunology field. It brings several research articles in which the immune background of bone tissue disorders, as well as the behavior of orthopedic implants important for the locomotor system, was investigated.

Osteoporosis is the most prevalent metabolic skeletal disease and public health problem, characterized by decreased bone mass and increased risk of fractures. Basically, osteoporosis reflects a state of unbalance between structural and biological demands for calcium and phosphate during inflammatory state. The aging-associated chronic low-grade inflammatory state affects osteoporotic bone development. A temporal framework between inflammation and osteoporosis appears to be important in aging, menopause, pregnancy, transplantation, and steroid applications. Importantly, the concordance of osteoporosis and inflammation is regulated by inflammatory mediators [1]. In this special issue, one of the aims was to reveal the prevalence of rare hematologic disease, systemic mastocytosis in patients with unexplained osteoporosis to identify whether mastocytosis is a plausible cause of secondary osteoporosis. Unlike primary osteoporosis, associated with aging, secondary osteoporosis is usually related to other diseases and drugs. Systemic mastocytosis was characterized by a clonal proliferation of neoplastic mast cells which can occur in the bone marrow. It is suggested that hypertryptasemia (high serum levels of mastocyte cell-specific serine protease tryptase) can be a useful marker of mastocyte-cell related diseases, such as bone fragility and secondary osteoporosis.

In the manuscript published in this special issue, a rat model was utilized to reveal the significance of the interaction of estrogen deficiency and hyperglycemia in the skeletal system. In this work, bilateral ovariectomy was applied to induce estrogen deficiency, while diabetes mellitus was established by single streptozotocin administration. Interestingly, measurement of several cytokines in the circulation revealed only one bioactive molecule, the chemokine regulated upon activation, normal T cell expressed and presumably secreted (RANTES), which is found to be significantly affected by both ovariectomy and diabetes mellitus in rats. Serum levels of chemokine RANTES, secreted by osteoblasts and osteoclasts in bone, were increased mainly in the ovariectomized diabetic rats. Results of this study suggest that the effects on bone microarchitecture and inflammation induced by hyperglycemia in rats were only slightly moderated by estrogen deficiency [2].

Osteonecrosis occurs when reduced blood flow to bone causes death of bone and marrow cells. Glucocorticoid and alcohol usage may result in osteonecrosis which can affect almost any bone of the body, where the hips, knees, and shoulders are the most common affected sites. Vascular occlusions, low-grade chronic inflammation, poor oxygen, and nutrient supply to the affected bone region are considered as major etiological indicators. Osteonecrosis-related chronic inflammation, including accumulation of activated neutrophils, macrophages, and T cells, is followed by the increased production of inflammatory mediators. Macrophages and neutrophils produce reactive oxygen and nitrogen species with proinflammatory activities. Another work published in this special issue explored the association of T cell populations (and their phenotype) in the pathophysiology of osteonecrosis that affects patients with sickle cell disease. Sickle cell disease is caused by a point mutation in the *β*-globin gene, resulting in the production of abnormal hemoglobin, deformed erythrocytes, and finally, inflammation-associated hemolysis and vascular occlusions. Since osteonecrosis is clinical complication of sickle cell disease, in this study, T cells were analyzed in human peripheral blood and bone marrow aspirates of healthy and sickle cell disease patients. This study reported the increased proportion of proinflammatory cytokine-producing CD4^+^ T cells in the bone marrow of sickle disease patients. From collected observations, it is suggested that these T cell populations may contribute to chronic inflammation and the pathophysiology of osteonecrosis in sickle cell disease [3].

Artificial joint replacement is the current clinical gold standard treatment for destructive joint diseases. Previous *in vitro* and *in vivo* experiments, as well as clinical studies reported that byproducts from joint replacements can induce chronic low-grade inflammation resulting in periprosthetic osteolysis, aseptic loosening, and prosthesis failure. Inflammatory insults in peri-implant areas, caused by the wear particles, provoke osteoclast activation at the bone–implant interface and initiate bone resorption. In awareness of the need of improved approaches for the treatment of aseptic loosening, the effect of tetrandrine on inflammatory osteolysis in a titanium particle-induced inflammatory osteolysis mouse model was investigated as well. It is found that tetrandrine inhibits the expression of proinflammatory cytokines, TNF-*α*, IL-1*β*, and IL-6, as well as receptor activator for nuclear factor-*κ*B ligand- (RANKL-) induced osteoclast formation and bone resorption [4]. Observed data suggest tetrandrine as reasonable treatment option for osteolytic peri-implant regions. Another study explored *in vitro* effects of tantalum and titanium dioxide nanoparticles on a macrophage cell line model. Although the therapeutic potential of tantalum-based implants for total joint replacement is well described, the effects of wear particles of tantalum implants on peri-implant cells and their potential contribution to aseptic implant loosening are not revealed. This is of particular importance since macrophage polarization initiated inflammatory signaling and osteoclastogenesis represents the main control point for cell death in peri-implant regions. This study reported a reduced potential of tantalum nanoparticles to induce TNF*α* and IL-1*β* as well as reactive oxygen species by macrophages when compared to titanium dioxide particles, suggesting that tantalum-based implants might be a more rational therapy option with lower risk to provoke aseptic loosening, as previously described [5].

We hope that this special issue provides new interesting insights on the complex immune background of bone diseases, as well as their significance in designing optimal biomaterials for orthopedic implants. In addition, we believe that this issue opens new questions which will be resolved in future fundamental and clinical studies.
